# Successful pregnancy following two-stage hysteroscopic myomectomy in women with diffuse uterine leiomyomatosis: a case report and literature review

**DOI:** 10.3389/fmed.2026.1762409

**Published:** 2026-04-10

**Authors:** Hui Yan

**Affiliations:** Department of Obstetrics and Gynecology, The First Affiliated Hospital of Ningbo University, Ningbo, Zhejiang, China

**Keywords:** diffuse uterine leiomyomatosis, fertility preservation, high-intensity focused ultrasound, hysteroscopy, uterine reconstruction

## Abstract

Diffuse uterine leiomyomatosis is frequently misdiagnosed, highlighting the need to improve awareness and understanding of this rare condition. We report a case of a 29-year-old nulliparous woman presenting with menorrhagia who was diagnosed with diffuse uterine leiomyomatosis and underwent a two-stage hysteroscopic myomectomy. She subsequently conceived naturally and delivered a healthy full-term infant. In addition, we review the existing literature on diffuse uterine leiomyomatosis to enhance its diagnosis and management.

## 1 Introduction

Diffuse uterine leiomyomatosis (DUL) is a rare benign condition that primarily affects women of reproductive age. It is characterized by the following: (1) numerous small (0.5–3 cm), poorly defined myomas; (2) involvement of the entire myometrium; and (3) symmetrical enlargement of the uterus (1). DUL is frequently misdiagnosed as multiple uterine fibroids or adenomyosis, as these conditions share clinical features such as menorrhagia, anemia, and uterine enlargement. Since most patients with DUL are of childbearing age and have fertility requirements, timely diagnosis and appropriate management are particularly important. To share our experience in diagnosis and management, we present a case of a 29-year-old women with DUL who conceived naturally and delivered successfully following a two-stage hysteroscopic myomectomy (HM).

## 2 Case description

A 29-year-old nulligravid woman (G0P0) with a desire for future fertility presented to our hospital in March 2024 with menorrhagia. A 2-cm uterine fibroid was detected by transvaginal ultrasonography in April 2020 during a routine physical examination. At that time, her menstrual cycle was normal, and she was advised to undergo regular follow-ups.

On admission, she reported experiencing progressively severe menorrhagia since March 2023. Her menstrual cycles wew regular (25-day cycle, 6-day duration) with occasional mild dysmenorrhea. She had no significant medical or surgical history and no family history of malignancy. She had one brother and one sister, and neither her mother nor sister had a history of uterine fibroids. The menorrhagia had caused anemia, with a nadir hemoglobin level of 5.7 g/L. Anemia was treated with iron supplementation, and her hemoglobin level was 8.9 g/L at the time of admission. Laboratory tests domonstrated normal coagulation function, anemia on complete blood count, negative serum *β*-human chorionic gonadotropin, normal reproductive hormone levels, and normal female tumor markers. Magnetic resonance imaging (MRI) was not performed due to prolonged waiting times. Three-dimensional transvaginal ultrasonography revealed a uterus measuring 5.5 × 5.7 × 6.7 cm. The myometrium contained multiple round lesions of varying sizes, most in contact with the endometrium. The largest lesion measured 3.7 × 2.4 × 3.5 cm, the endometrial thickness was approximately 0.6 cm, and a 3-cm chocolate cyst was observed in the left adnexal region. The patient was initially diagnosed with multiple myomas and endometriotic cysts. Laparoscopy combined with a hysteroscopy was performed to remove the myomas and ovarian cyst. Due to multiple uterine fibroids and anemia, three units of packed red blood cells (PRBCs) were prepared preoperatively; however, no transfusion was required before surgery.

**Figure 1:**
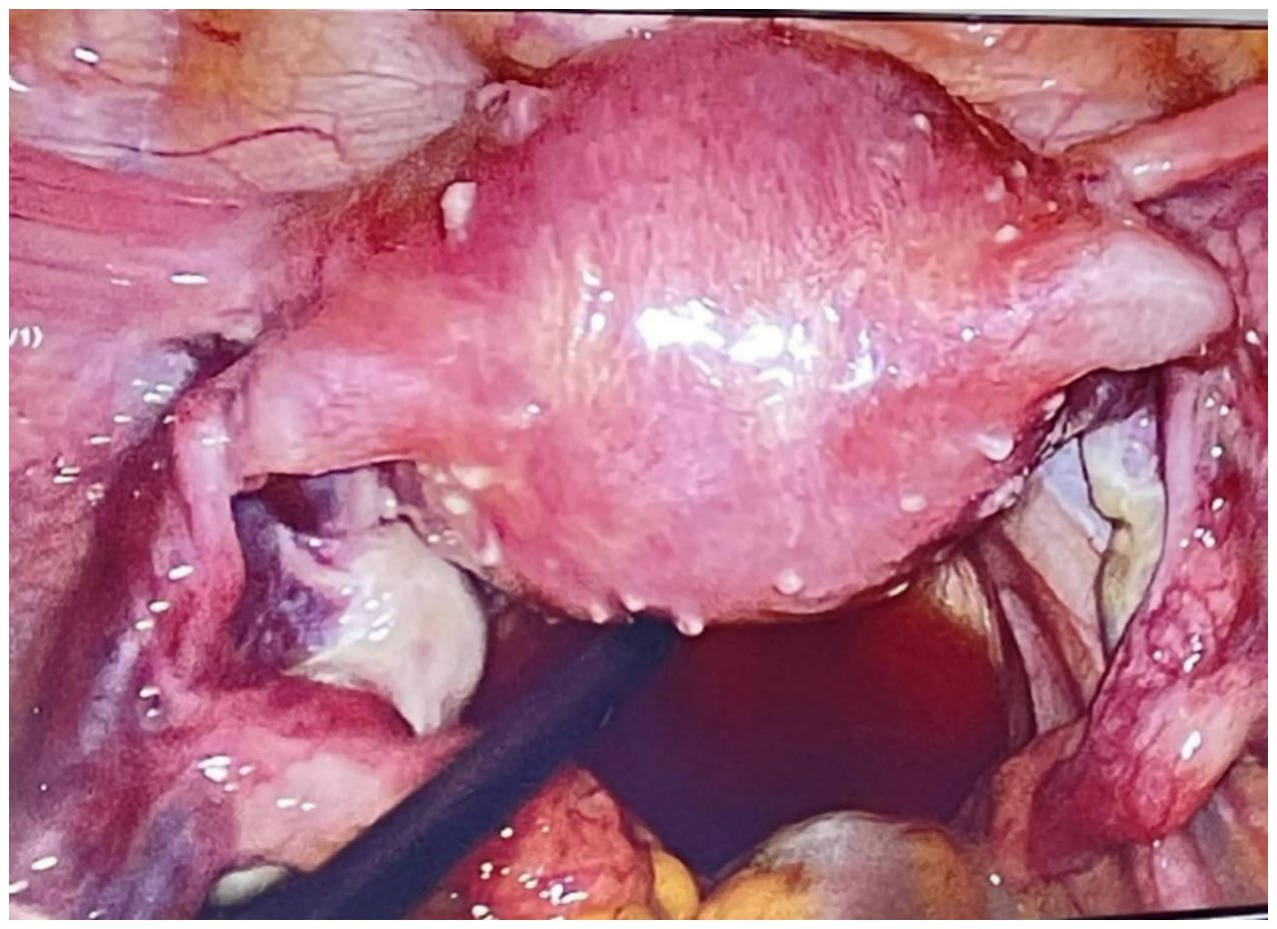
Laparoscopic view showing numerous rice-grain-sized fibroids covering the surface of the uterus.

**Figure 2:**
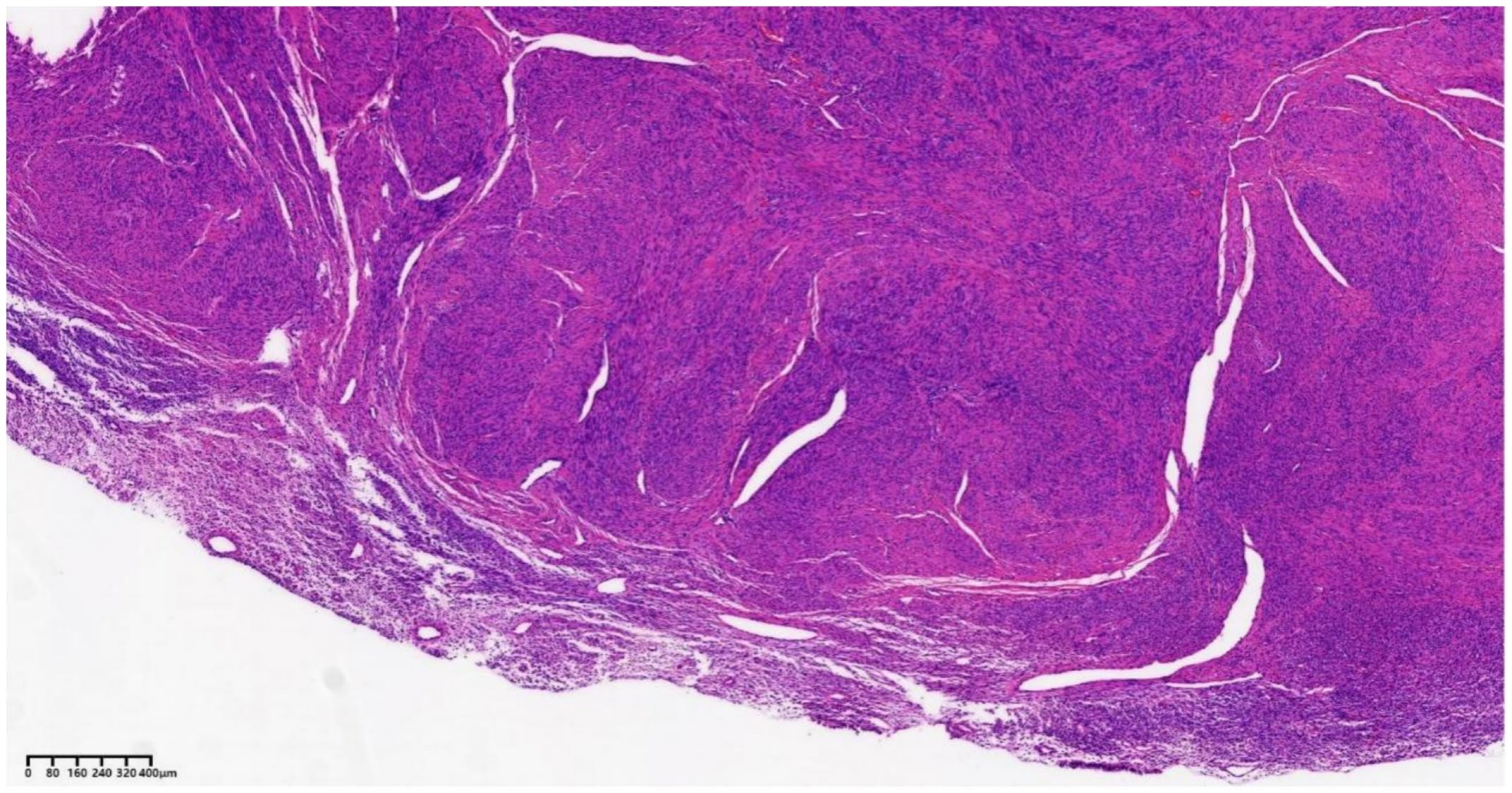
Histopathology of the nodules on the uterine surface confirmed uterine fibroids removed during the first surgery.

**Figure 3:**
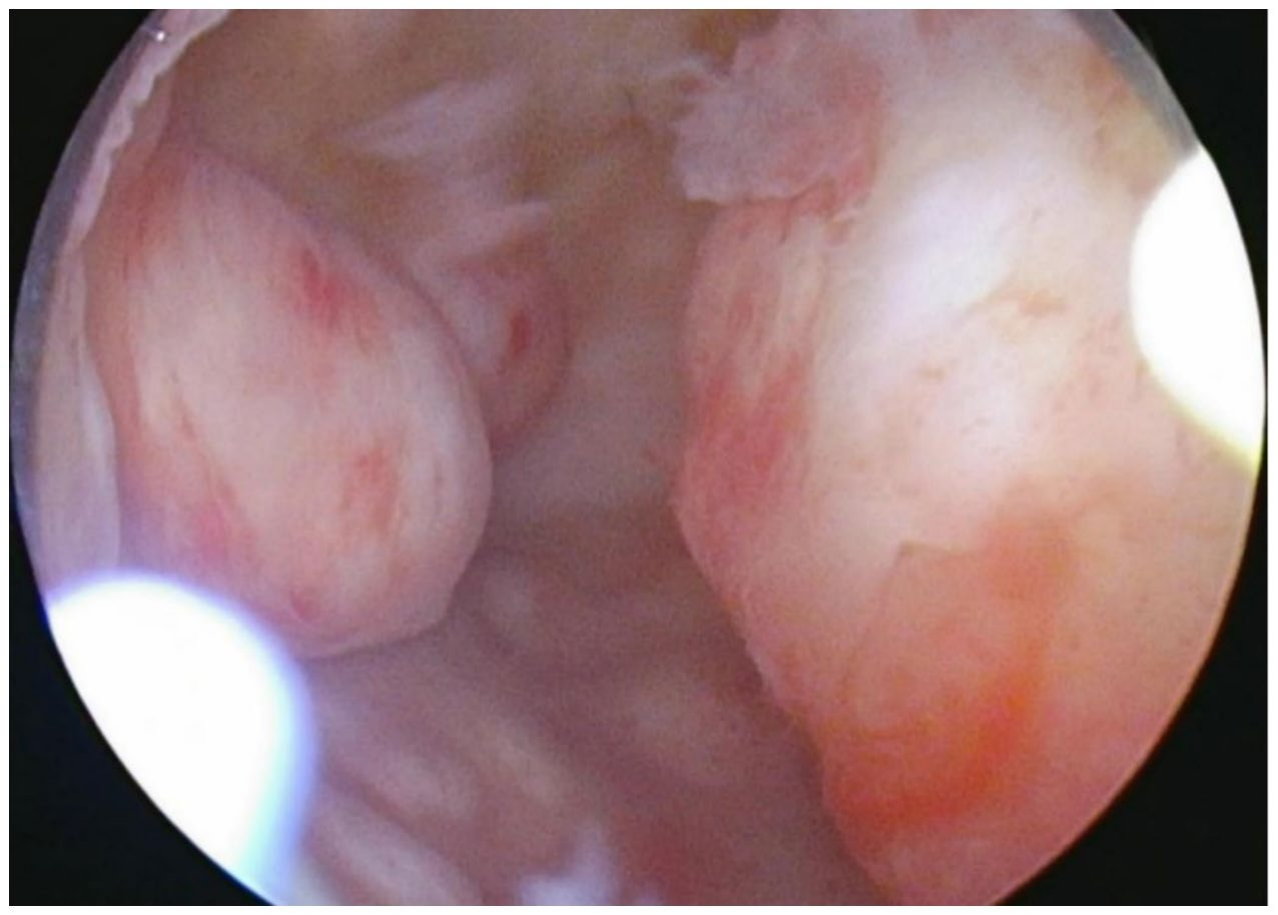
Hysteroscopic view showing the walls of the uterine cavity largely covered with protrusions of varying sizes, with a smooth endometrium and the openings of both fallopian tubes visible.

Laparoscopy revealed numerous rice-sized nodules scattered across the uterine surface (Figure 1), and no additional large uterine fibroids were identified. There were no significant adhesions between the ovaries and adjacent structures. The morphology of both fallopian tubes was preserved and appeared normal. The right ovary was intact, with no evidence of adhesions or cystic lesions. Meticulous dissection was performed to separate the left ovarian cyst, which was filled with chocolate-like fluid, from the surrounding normal ovarian parenchyma, and the cyst was completely excised. Several rice-sized nodules were resected for histopathological evaluation, and were confirmed to be uterine fibroids (Figure 2). Under hysteroscopy, the uterine cavity was distorted; the anterior, posterior, and lateral walls were covered with type 0–3 fibroids of varying sizes (Figure 3), and the opening of the fallopian tubes were visible bilaterally.

To prevent intrauterine adhesions (IUAs), a two-stage HM was scheduled. A 26-Fr hysteroscope with a with a 30° viewing angle (Olympus Co., Tokyo, Japan) was used to resect the myomas while preserving the pseudocapsule and avoiding damage to the surrounding myometrium. The electrosurgical loop was typically set at 100–120 W in cutting mode and 60–75 W in coagulation mode during the procedure. Normal saline (0.9%) was used as the distention medium. A small incision was made over each fibroid to expose its pseudocapsule, and resection was carefully performed within the pseudocapsule, as described in our previous study (2). Intraoperatively, a single dose of furosemide was administered to prevent fluid overload, and 20 unites oxytocin were used to facilitate uterine contraction. The total operative duration was 90 min, with an estimated intraoperative blood loss of approximately 50 mL. No perioperative blood transfusion was required.

Myomas on the anterior and posterior opposing walls were not removed in the same procedure and were deferred to a second-stage resection. In the initial surgery, two type 0 myomas, three type 1 myomas, and six type 2 myomas which located in the posterior and lateral uterine wall were completely removed. The myomas on the anterior uterine wall and those remaining on the lateral uterine wall were scheduled for second-stage resection. Histopathological examination revealed submucosal myomas and an ovarian endometriotic cyst. The patient was diagnosed with DUL accompanied by endometriosis and was scheduled to for uterine reconstruction 1 month later.

**Figure 4:**
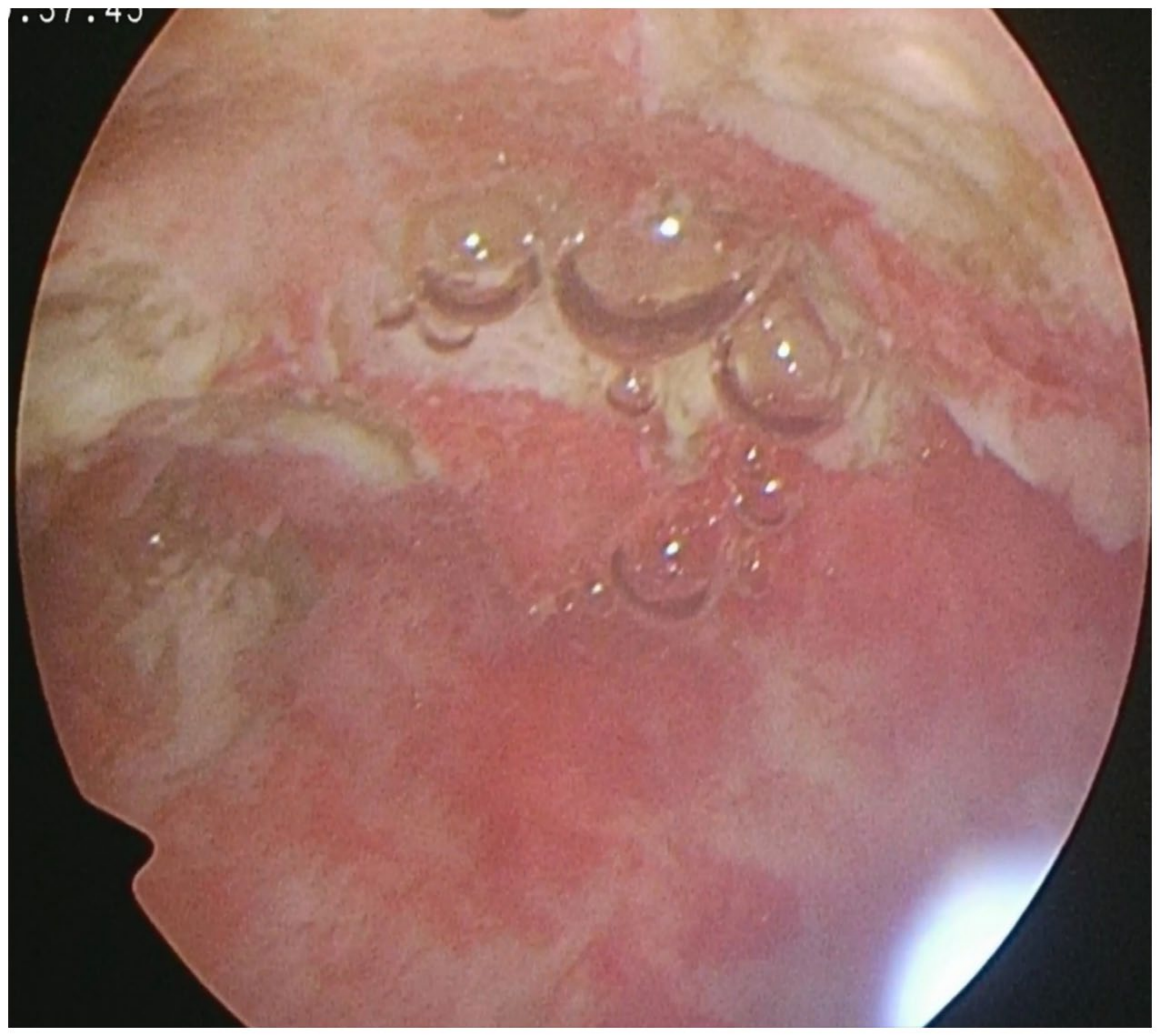
Hysteroscopic view of a clean uterine cavity following the second hysteroscopic myomectomy.

During the second hysteroscopy, the uterine cavity was clear, without any IUAs, and multiple type 1–3 fibroids remaining from the previous surgery increased in size and were promptly resected (Figure 4). The patient was discharged and administered 3.75 mg of gonadotropin-releasing hormone antagonist (GnRH-a) every 4 weeks for 2 months. She spontaneously conceived following her first menstrual period but subsequently underwent a medical abortion due to poor embryonic development. Six months later, she conceived spontaneously again and delivered a term male infant by cesarean section at 38 weeks of gestation in December 2025. During the cesarean section, two submucous fibroids, each approximately 1 cm in diameter, were simultaneously resected and confirmed by histopathological examination and which confirmed uterine fibroids. The placenta was manually removed smoothly, with no signs of postpartum hemorrhage. Since the first abortion, transvaginal ultrasonography revealed recurrent uterine fibroids, the largest measuring approximately 1 cm, which did not show any enlargement throughout the pregnancy. Prenatal ultrasound demonstrated a normally positioned placenta with no evidence of placenta accreta. On 15 January 2026, postpartum follow-up transvaginal ultrasound showed satisfactory uterine involution and a submucous myoma measuring 7 × 4 mm. As the patient was breastfeeding, no uterine fibroid-modulating agents were administered to inhibit tumor growth.

## 3 Discussion

A comprehensive literature search was conducted in the following electronic databases from their inception to 1 October 2025 to identify all relevant studies on diffuse uterine leiomyomatosis (DUL). PubMed and Web of Science were searched to ensure comprehensive literature retrieval. The MeSH Terms used included Uterine Leiomyoma, Diffuse Leiomyomatosis, Hysteroscopy, and Myomectomy. Case reports, retrospective studies, prospective studies, and review articles related to DUL were included, whereas studies unrelated to DUL were excluded.

DUL is a rare and distinct form of uterine leiomyoma, characterized by numerous poorly circumscribed hypercellular nodules of bland smooth muscle cells without cytologic atypia, diffusely involving the myometrium. The etiology and pathogenesis of DUL remain unknown. Because DUL lacks specific clinical manifestations, it is frequently misdiagnosed preoperatively as multiple uterine leiomyomas. Therefore, DUL should be considered when ultrasonography or MRI suggests multiple uterine leiomyomas. As with many other gynecological disorders, common symptoms of DUL include menorrhagia and infertility. Ren et al. (3) reported a case in which transabdominal myomectomy was performed due to a misdiagnosis of multiple myomas. Intraoperative blood loss was 1,800 mL, and menorrhagia recurred after six courses of GnRH-a. Tam (4) presented a 31-year-old woman diagnosed with leiomyomas and abnormal uterine bleeding (AUB) who underwent two hysteroscopic and robot-assisted myomectomy procedures within 1 year. Her treatment failed, and a hysterectomy was ultimately performed due to persistent AUB. Seven months after surgery, uterine histopathology confirmed DUL.

Although ultrasonography is the first-line imaging modality for evaluating the pelvic cavity and uterine or ovarian disorders, MRI is superior for diagnosing DUL, because it provides excellent soft-tissue contrast and enables detailed mapping and evaluation of the nodules, including their number, size, and location within the uterine layers. This information is essential for establishing a definitive diagnosis and planning effective treatment. On MRI, DUL produces a characteristic “pebble-filled purse” appearance and symmetrical uterine enlargement, caused by numerous small leiomyomas with indistinct margins that merge through coalescing fibers. Preoperative MRI is recommended when ultrasound assessment is limited by the presence of numerous leiomyomas (5). DUL with disseminated peritoneal leiomyomatosis should also be considered in patients presenting with multiple uterine leiomyomas and additional masses in the parametrial or abdominal cavities on MRI (6).

Currently, hysterectomy is the only definitive treatment for women who do not wish to preserve fertility. For patients seeking fertility preservation, several alternative treatment modalities have been reported in the literature.

The novel transabdominal myomectomy technique, which allows removal of serosal, intramural, and mucosal myomas, involves a midline longitudinal uterine incision while preserving both uterine arteries. Transient occlusion of the uterine arteries is employed in this approach to reduce intraoperative bleeding (6, 7); however, it is not considered a first-line option for most patients. Uterine rupture during subsequent pregnancy has not been reported to date; nevertheless, further studies with larger cohorts and long-term follow-up are required to determine fertility outcomes.

Uterine artery embolization (UAE) has been reported as an effective treatment for reducing DUL-related symptoms and uterine volume. A retrospective study demonstrated that all seven patients with DUL-associated menorrhagia experienced symptomatic improvement at the mid-term follow-up after UAE. (8) The mean reduction in uterine volume reduction was 50.1%, and one patient conceived 5 months after the procedure (8).

High-intensity focused ultrasound (HIFU) is another uterus-conserving treatment that alleviates symptoms in patients with DUL. Chen et al. (9) first reported the case of a 38-year-old patient with DUL and a uterine size equivalent to 5 months’ gestation, who was treated with HIFU. Her menstrual volume, duration and cycles normalized, and no dysmenorrhea was observed after the third HIFU session. The uterine volume was reduced to 44% of its initial size. Pregnancy outcomes were not reported in this case. Another study involving eight patients with DUL treated with HIFU reported a mean uterine volume reduction of 67.6% after an average follow-up of 5.9 months. Zhang et al. (10) Anemia improved following relief from menorrhagia over a mean follow-up of 19.1 months (10). As six of the eight patients were unmarried and the remaining two had no immediate pregnancy plans, post-HIFU pregnancy outcomes were not recorded. Gong et al. (11) reported that three patients conceived between three and 11 months after two sessions of HIFU, and all of whom delivered healthy infants. They speculated that HIFU ablation may facilitate successful pregnancy in patients with DUL.

HM has been widely used in DUL to remove submucosal myomas and restore a normal endometrial cavity, serving as an alternative to extensive abdominal myomectomy for clearing intramural myomas. Good fertility outcomes following hysteroscopic resections of submucosal fibroids in patients with DUL have been reported in the literature (12, 13). For hysteroscopic myomectomy, strict control of operative duration and performance of intracapsular myoma resection are of critical importance. Prolonged surgery significantly increases the risk of fluid overload, which can result in severe complications. After incising the pseudocapsule, intravenous oxytocin was administered, and the uterine distension pressure was reduced to 100 mmHg. The combination of decreased intrauterine pressure and enhanced uterine contractions facilitates further protrusion of type 1–3 myomas into the uterine cavity, thereby shortening resection time. Intracapsular resection not only reduces intraoperative blood loss but also minimizes the risk of fluid overload. The pseudocapsule is relatively hypovascular, which decreases both bleeding and systemic absorption of distension fluid. Additionally, this technique reduces thermal damage to the surrounding endometrium. Minimizing IUAs after hysteroscopic myomectomy and reducing thermal damage to the endometrium have received increasing attention from surgeons. In 2014, Mazzon et al. (14) reported that “cold loop” HM may reduce the prevalence of IUAs. They employed a two-step “cold loop” HM procedure for DUL and observed no submucous myomas and a regular, synechiae-free uterine cavity on endoscopy (14). Hysteroscopic surgery using cold graspers combined with electric loop via the hysteroscopic endo-operative system (HEOS) has also been reported to preserve the uterus and effectively protect the endometrium from IUAs (15). Seven of the eight women (87.5%) conceived spontaneously postoperatively, with one patient experiencing a miscarriage during the second trimester (live birth rate, 6/8, 75%) following surgery using the HEOS. Only two mild cases of IUAs were identified after the first hysteroscopic procedure (15). In 2024, Zhang et al. (16) reported a “no-distension” HM using thoracic tissue forceps. The forceps allowed accurate grasping and peeling of fibroids under transvaginal ultrasound in a non-distended uterus; 38 uterine fibroids were successfully excised without complications; and no IUAs and residual myomas were observed during the follow-up hysteroscopy (16).

GnRH-a is a conservative treatment that plays an important role in managing DUL. It can be used alone or as an adjunct in combination with other treatment modalities for DUL. In one reported case, a patient with DUL and a uterus enlarged to the size of a 14-week pregnancy experienced normalization of uterine size and subsequently conceived after receiving intranasal buserelin acetate (900 μg/day), a GnRH-a, for 6 months (1). However, some patients with DUL exhibit a poor response to hormone therapy, according to the previous reports.

DUL is a subtype of uterine leiomyoma, and MRI is useful for pretreatment evaluation. Given the fertility demands of these patients, accurate diagnosis and assessment prior to surgery are essential.

Multiple fertility-preserving treatment modalities have been reported, primarily in case reports and case series; however, few large- scale, high-quality studies have clarified which approach is most effective for preserving fertility. No-energy hysteroscopic surgery has received some attention, but tailored management strategies for DUL should be developed based on a comprehensive understanding of this rare condition.

## Data Availability

The original contributions presented in the study are included in the article/supplementary material, further inquiries can be directed to the corresponding author.
